# Laminar Flow Attenuates Macrophage Migration Inhibitory Factor Expression in Endothelial Cells

**DOI:** 10.1038/s41598-018-20885-1

**Published:** 2018-02-05

**Authors:** Congzhen Qiao, Shengdi Li, Haocheng Lu, Fan Meng, Yanbo Fan, Yanhong Guo, Y. Eugene Chen, Jifeng Zhang

**Affiliations:** 1 Department of Pharmacology, University of Michigan Medical Center, Ann Arbor, Michigan 48109 USA; 2 Shanghai Jiao Tong University School of Medicine, Shanghai, 200025 China; 3 Key Lab of Computational Biology, CAS-MPG Partner Institute for Computational Biology, Shanghai Institutes for Biological Sciences, Chinese Academy of Sciences, Shanghai, 200031 China; 4 University of Chinese Academy of Sciences, Beijing, 100049 China; 5 Frankel Cardiovascular Center, Department of Internal Medicine, University of Michigan Medical Center, Ann Arbor, Michigan 48109 USA; 6 Department of Psychiatry and Molecular and Behavioral Neuroscience Institute University of Michigan Medical Center, Ann Arbor, Michigan 48109 USA

## Abstract

Macrophage migration inhibitory factor (MIF) is a non-canonical cytokine that is involved in multiple inflammatory diseases, including atherosclerosis. High MIF expression found in leukocytes which facilitates the initiation and progression of atherosclerosis. However, little is known about biomechanical forces in the induction of MIF in endothelial cells (ECs). Here, we show that laminar shear stress (LS) inhibits the expression of MIF in ECs. By profiling the whole transcriptome of human coronary artery ECs under different shear stress, we found that athero-protective LS attenuates the expression of MIF whereas pro-atherosclerotic oscillatory shear stress (OS) significantly increased the expression of MIF. *En face* staining of rabbit aorta revealed high MIF immunoreactivity in lesser curvature as well as arterial bifurcation areas where OS is predominant. Mechanistically, we found that Krüpple like factor 2 (KLF2) is required for inhibition of MIF expression in ECs in the context of shear stress. Knockdown of KLF2 abolishes LS-dependent MIF inhibition while overexpression of KLF2 significantly attenuated MIF expression. Overall, the present work showed that MIF is a shear stress-sensitive cytokine and is transcriptionally regulated by KLF2, suggesting that LS exerts its athero-protective effect in part by directly inhibiting pro-inflammatory MIF expression.

## Introduction

Atherosclerosis is the major cause of coronary artery disease as well as stroke, the top two killers worldwide^1^. Multiple lines of evidence demonstrate that endothelial dysfunction is fundamental to the process of atherosclerosis^2^. Once endothelial cells (ECs) become pathologically activated, pro-inflammatory factors such as tumor necrosis factor (TNF)-α, interleukin (IL)-1β^3^ and adhesive molecules, including E-selectin and vascular cell adhesion protein 1 (VCAM1), are up-regulated in ECs facilitating leukocytes recruitment^4^ followed by accumulation of cholesterol-bound lipoprotein^5^ and signaling resulting in aberrant proliferation of smooth muscle cells^6^, initiating the formation of atherosclerotic lesions. Despite the multifactorial causes of endothelial dysfunction, low or oscillatory shear stress (OS) generated by disturbed blood flow contributes significantly to the development of atherosclerosis^7,8^. OS occurs in arterial regions of lesser curvature as well as arterial bifurcation areas^9^. OS-induced biomechanical stimulation compromises endothelial function, leading to atherogenesis^7^, highly consistent with the unique regional distribution of atherosclerotic lesions^10^. Human ultrasonographic imaging illustrates that the local shear stress magnitude inversely correlates with carotid artery intima-media thickness^11^. Animal studies have revealed a causal relationship between OS and atherosclerosis by demonstrating that OS initiates the otherwise atherosclerotic-resistant common carotid arterial intima into developing atherosclerotic lesion by perivascular constriction^12^. Understanding the mechanisms of biomechanical activation of endothelial function is a key to the treatment of atherosclerosis.

Given the high atherosclerotic susceptibility in OS-exposed areas, studies have demonstrated that pro-inflammatory factors induced by OS are actively involved in the process of atherosclerosis^8^. Macrophage migration inhibitory factor (MIF), also known as glycosylation-inhibiting factor (GIF), is an important regulator of innate immunity and is also a CXCR-non-canonical cytokine that has been positively associated to inflammation and atherosclerosis^13^. High MIF expression level in atherosclerotic lesions is found in animal models^14^ as well as human subjects^15^. Studies have further demonstrated that blockage of MIF in macrophages reduces the formation of atherosclerotic lesions in atherosclerotic animal models^16,17^. Nonetheless, the endothelium is as a significant source of MIF as well^18^ and EC MIF plays a central role in atheroma development and outcomes^19^. However, the mechanisms regulating MIF expression in ECs remain elusive.

We sought to investigate the role of biomechanical activation in endothelial cell biology by profiling the whole transcriptome of human coronary artery endothelial cells (HCAECs) under different shear stresses. We found that MIF is sensitive to biomechanical stimulation. MIF expression in HCAECs was significantly reduced under physiological LS in comparison to OS and static culture conditions. Thus, we hypothesized that EC-specific MIF, a pro-inflammatory cytokine, contributes to the process of atherosclerosis with its atherogenic effect naturally inhibited by LS *in vivo*. The present work provides direct evidence to show that biomechanical activation is essential in regulating the pro-atherosclerotic cytokine MIF. In addition, we found that LS-dependent MIF inhibition was mediated by increasing KLF2 expression in ECs. This natural protective mechanism of LS may shed light towards the development of anti-atherosclerotic therapeutics.

## Results

### Identification of shear stress sensitive genes in HCAECs

**Figure 1:**
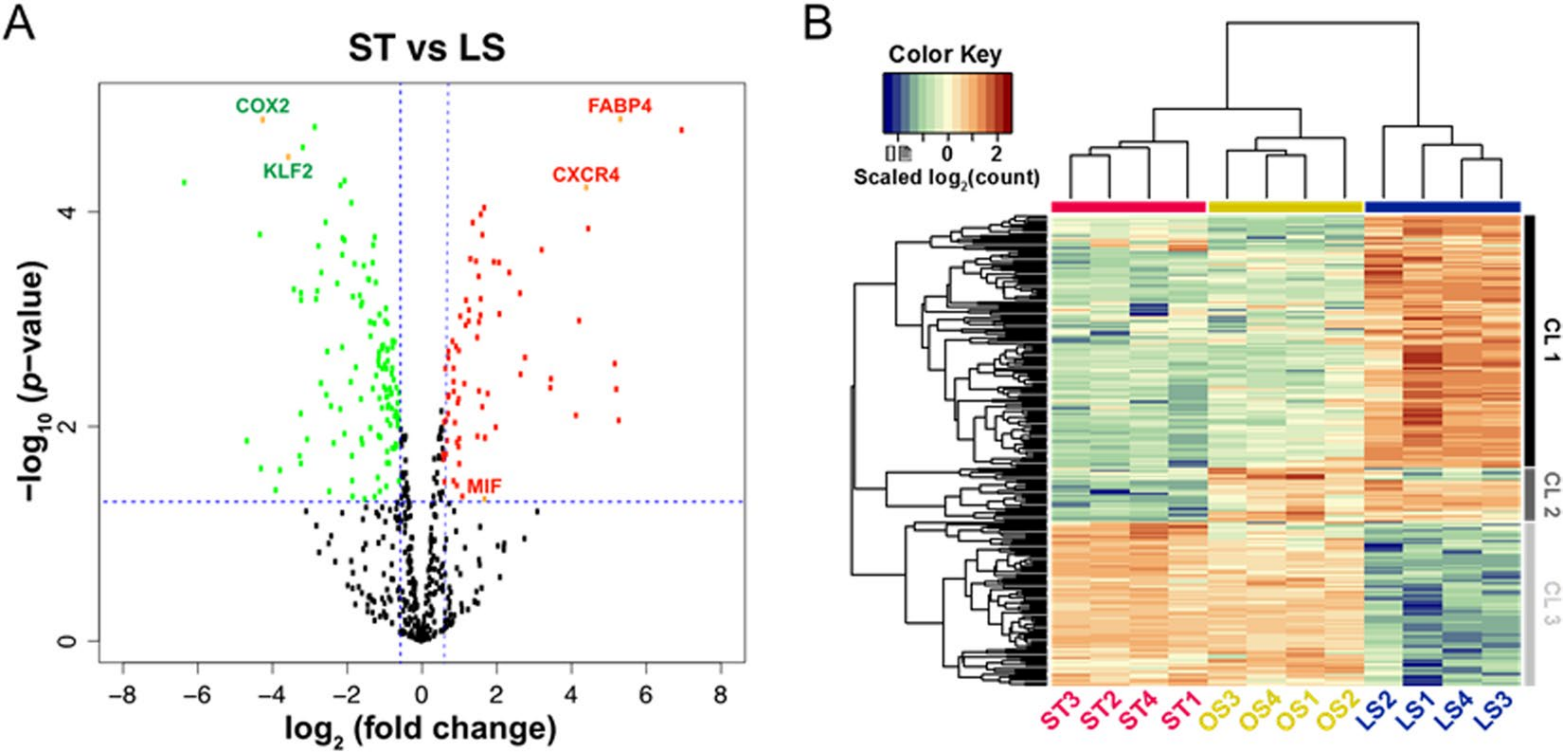
Modulation of genes in the inflammation category in response to different types of shear stress. (**A**) Volcano plot of all the inflammation-related genes detected in HCAECs in ST and LS conditions. y-axis is the negative log_10_ of *p* value; x-axis is the log_2_ of fold change. Green dots represent genes downregulated in HCAECs under ST condition with significant *p* value; red dots represent genes upregulated in HCAECs under ST condition with significant *p* value; black dots represent genes without significant changes. (**B**) A heat map of all the inflammation-related genes that are differentially expressed in HCAECs under different shear stress conditions. CL1, CL2 and CL3 represent gene cluster one, two and three respectively, which were determined based on similarity of expression profiles among three conditions. CL1 is comprised of genes that are stimulated by LS condition, while genes in CL3 are more active under ST and OS. CL2 contains a small group of genes that shows up-regulation in OS and LS, but not ST.

To investigate the effect of biomechanical activation on the endothelial transcriptomic profile and search for novel shear stress sensitive transcripts, we first employed RNA-seq to survey the whole transcriptome of HCAECs under conditions of LS, OS and ST for 24 hours. There were 16,313 genes with at least one count per aligned million reads (>=1 fpm) detected in this study, over 6,000 of which were differentially expressed in response to different types of shear stress. In the present work, we focused on the shear stress-induced inflammatory genes in ECs considering that atherosclerosis is well-recognized as a chronic inflammatory disease^20^. Genes that are documented in the inflammation category by Gene Ontology function annotation are presented as a volcano plot (Fig. 1A). Notably, pro-inflammatory factors such as fatty acid binding protein 4 (FABP4), C-X-C chemokine receptor type 4 (CXCR4) were significantly upregulated whereas anti-inflammatory factors including cyclooxygenase 2 (COX2) and Krüppel-like factor 2 (KLF2) were markedly down-regulated in ECs under ST condition compared to those under LS condition (Fig. 1A). A heat map of all differentially expressed genes in inflammation category is shown in Fig. 1B, revealed that gene expression pattern in ECs under LS condition were completely different from those under OS and ST conditions^21^.

### Pro-inflammatory cytokine MIF is sensitive to shear stress and co-localizes with OS in the arterial tree

**Figure 2:**
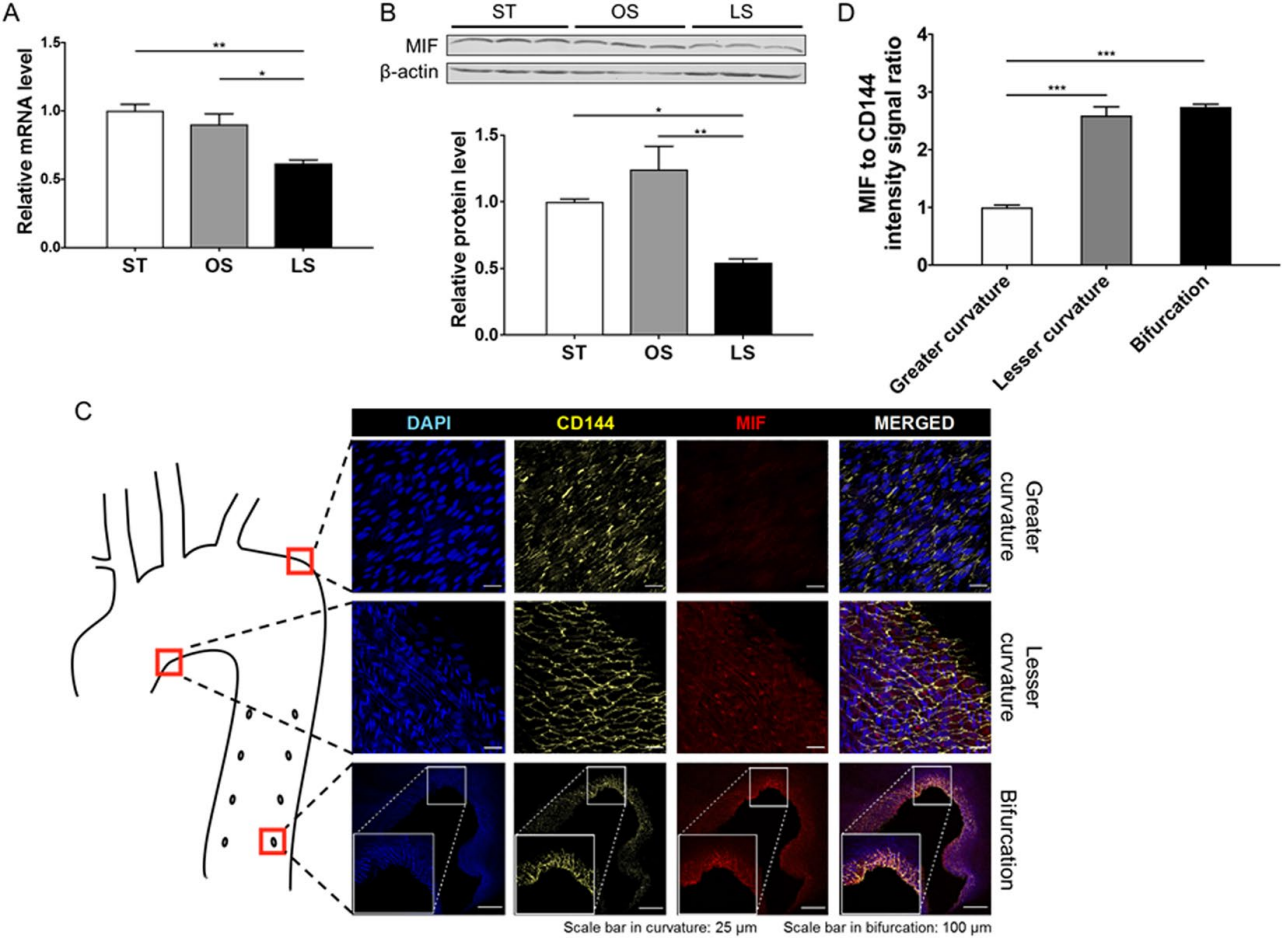
MIF expression in ECs is regulated by shear stress. The transcript level of MIF detected by qPCR (**A**) in human coronary artery endothelial cells (HCAECs) after 24-hour exposure to static (ST) culture, oscillatory shear stress (OS) and laminar shear stress (LS) (n = 4). Representative immunoblot (top) band intensity quantification (bottom) (**B**) of intracellular MIF protein levels in human umbilical endothelial cells (HUVECs) after 24-hour exposure to ST, OS and LS (n = 3). In all bar graphs, data are shown as mean ± SEM. *P < 0.05, **P < 0.01. (**C**) En face immunostaining with anti-CD144 (vascular endothelial cadherin, yellow) and anti-MIF (red) and counterstaining with DAPI (nuclear, blue) to show relative MIF protein abundance in the different parts of the rabbit aorta (location is shown in the Cartoon). MIF is highly expressed in lesser curvature as well as bifurcation areas. Data are representative of four independent experiments. Scale bar in upper 2 panels: 25 μm; Scale bar in the bottom panel: 100 μm. (**D**) Quantification of the data using Image J shows MIF intensity (***P < 0.001 vs greater curvature; n = 4).

Interestingly, we found that the pro-inflammatory factor MIF that is a novel shear stress sensitive gene based on our RNA-seq data (Fig. 1A). MIF has been implicated in multiple inflammatory diseases including atherosclerosis^13–15^. Transcript levels of MIF were confirmed by qPCR (Fig. 2A) and showed a nearly 50% decrease in HCAECs under LS condition compared to ST or OS conditions. To assess MIF expression in other ECs, human umbilical vein ECs (HUVECs) were used to demonstrate reduced protein levels of MIF under LS (Fig. 2B). Next, we asked if MIF expression is associated with the flow pattern *in vivo*, which varies spatially in the arterial tree. Aortas from New Zealand white rabbits fed a chow diet were harvested for *en face* immunostaining. Consistent with the *in vitro* data, a high level of MIF immunoreactivity was detected in OS-exposed regions including lesser curvature as well as bifurcation areas while the level of MIF was barely detectable in straight descending aorta subjected to LS-like forces (Fig. 2C,D). Taken together, MIF is a novel shear stress-sensitive cytokine, the expression of which is high in atherosclerotic-prone areas and is low in athero-resistant segments *in vivo*, suggesting a potential role of MIF in OS-induced endothelial dysfunction.

### MIF is transcriptionally regulated by KLF2

**Figure 3:**
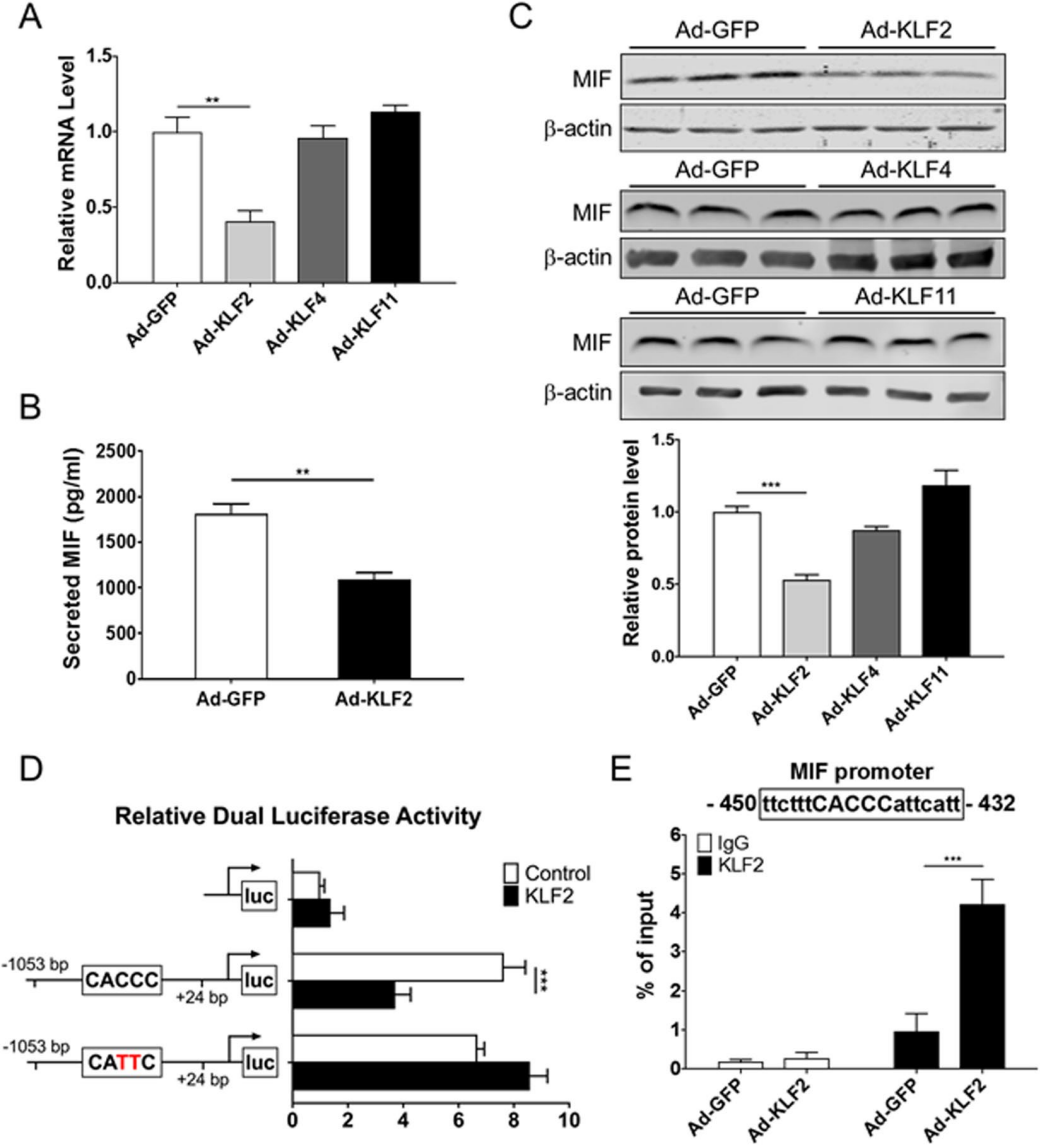
MIF expression is transcriptionally regulated by KLF2. (**A**) MIF mRNA level in HUVECs determined by qPCR after 48-hour adenovirus-mediated overexpression of KLF2, KLF4 and KLF11 (n = 3). mRNA expression was normalized to HPRT1 mRNA. (**B**) A total of 2 × 10^5^ cells were counted and seeded into each well for the following MIF ELISA assay. HUVECs were infected with Ad-GFP, Ad-KLF2 for 2 h followed by incubation in fresh medium for 48 h. MIF secreted by HUVECs was measured by ELISA after another 48-hour incubation in serum-free medium (n = 6). (**C**) Representative Western blotting (top) and the corresponding quantitative analysis of band intensity (bottom) show the expression of intracellular MIF protein relative to the loading control β-actin in ECs overexpressing KLF2, KLF4, KLF11 or GFP (n = 3). (**D**) Dual luciferase activity was assessed in Ad293 using the 1-kB MIF promoter driven Luciferase construct and the mutated one in combination with expression plasmids for KLF2. Mutation of the indicated binding sites was performed using the Site-Directed Mutagenesis kit. Data represent relative luciferase activity (normalized to *Renilla* luciferase), n = 6 per group. (**E**) HUVECs were infected with adenovirus-KLF2 or -GFP for 72 hours. ChIP assay was performed using a rabbit antibody against KLF2 and equivalent amount of rabbit IgG as control. The binding of KLF2 to the MIF promoter was determined by qPCR. Data are shown as mean ± SEM. **P < 0.01, ***P < 0.001.

Given the high expression of the pro-inflammatory MIF in OS predominant areas, we asked if it would be regulated by shear stress-sensitive transcription factors in ECs. The key shear stress-sensitive transcription factor KLF2^22^ functions as a master regulator of EC proliferation, thrombosis and inflammation. Thus, we first surveyed the effects of KLF family members that are also involved in inflammation on MIF expression. In addition to KLF2, KLF4 and KLF11 have been implicated in inflammation in ECs as well^23–25^. Thus, we sought to detect MIF transcript levels in adenovirus-infected HUVECs overexpressing KLF2, KLF4 and KLF11, respectively, for 48 hours. MIF mRNA levels were down-regulated only by KLF2 overexpression while no significant difference was observed in KLF4- and KLF11-overexpressing ECs (Fig. 3A). Human MIF ELISA assay was used to examine the secreted MIF after 48-hour incubation in serum-free medium. The level of secreted MIF was significantly lower in KLF2 overexpressing cells than the GFP control (Fig. 3B). The intracellular protein level of MIF was significantly reduced in ECs infected with adenovirus-KLF2 compared to the one with adenovirus-GFP infection by Western blotting (Fig. 3C). There was no significant difference of MIF protein level in ECs infected with adenovirus-KLF4 and -KLF11 (Fig. 3C).

To determine a potential transcriptional regulatory effect of KLF2 in inhibition of MIF expression, luciferase reporter assay and chromatin immunoprecipitation (ChIP) were performed in HUVEC. We found that a reporter containing a putative KLF2 binding site is enough to inhibit the basal expression of a MIF luciferase reporter *in vitro* by KLF2 overexpression compared to GFP (Fig. 3D). Mutation of the binding site restored the expression of luciferase to levels comparable to the GFP expressing adenovirus (Fig. 3D). The KLF2 binding site was shown to be functional by ChIP assay. We demonstrated that KLF2 directly binds to the MIF promoter region where contains the specific binding site (Fig. 3E) in the same conditions that inhibit MIF expression in these cells (Fig. 3A,B and C).

### KLF2 knockdown in ECs attenuates LS-dependent MIF inhibition

**Figure 4:**
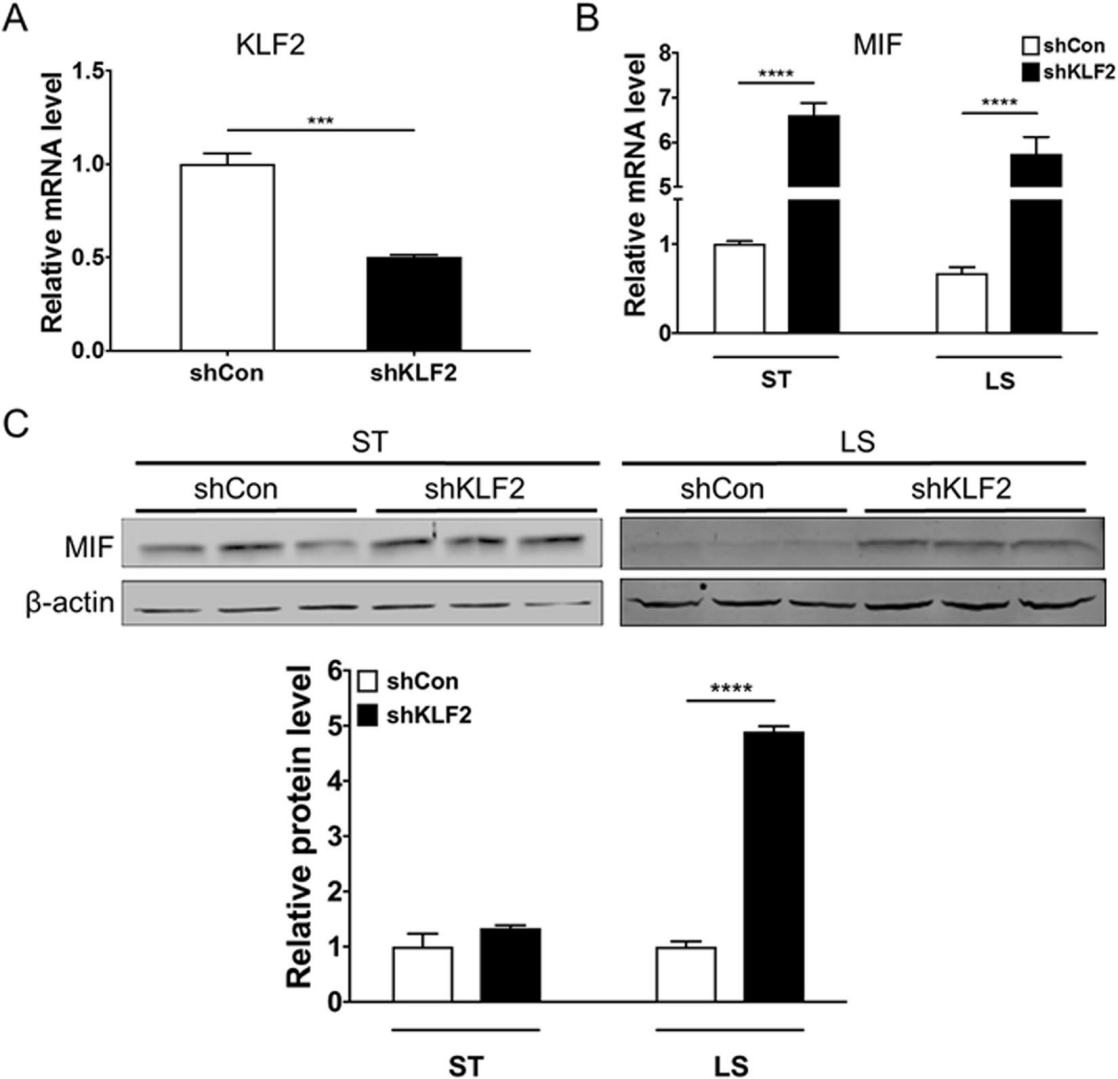
Knockdown of KLF2 prevents LS-induced reduction of MIF. HUVECs were infected with adenovirus-shKLF2 or -shControl for 48 hours before LS stimulation for 24 hours. The RNA expression of KLF2 (**A**) and MIF (**B**) were detected by qPCR. (**C**) Representative Western blot of intracellular MIF and β-actin in ECs infected with adenovirus-shKLF2 or -shControl under static culture (ST) or LS conditions. Data shown are presented as mean ± SEM. ***P < 0.001, ****P < 0.0001.

To test the effect of KLF2 in LS-dependent inhibition of MIF, HUVECs were infected with adenovirus-shKLF2 to down-regulate the endogenous KLF2 levels and were then exposed to LS for 24 hours. The relative KLF2 mRNA levels were reduced by about 50% in HUVECs under LS condition (Fig. 4A) and endogenous MIF was significantly increased in the KLF2 knockdown group at both the transcript (Fig. 4B) and protein level (Fig. 4C) under both the ST and LS conditions. Of note, there was no statistical significance in MIF expression between KLF2 knockdown group and control under ST, whereas a significant increase of MIF in KLF2 knockdown group under LS (Fig. 4C).

## Discussion

Cardiovascular disease is the top killer worldwide, contributing to around 1/3 of mortality^26^. Atherosclerosis is recognized as the fundamental source of ischemic heart disease and stroke^27^. It is mainly characterized by the deposition of circulating lipids, the infiltration of leukocytes and the aberrant proliferation and apoptosis of ECs and SMCs in the vasculature. A great number of studies focus on the causes and pathogenesis of atherosclerosis, with variations in the hemodynamic forces generated by blood flow long recognized as a primary driver of atherosclerosis and responsible for the non-random topography of the lesions^10^. The whole transcriptomic profile in ECs under ST, OS and LS conditions described here revealed a great number of novel shear sensitive genes that have not been previously reported.

Since inflammation is a primary driver of atherosclerosis, here we focused our RNA-seq data analysis on the inflammatory pathways. We uncovered that endothelial MIF is a novel shear stress sensitive cytokine, thus adding a new perspective of its role in relation to early development of atherosclerosis. In fact, multiple lines of evidence established that MIF may be a pro-atherogenic cytokine that positively correlates with increased vessel wall thickening and lipid deposition in the aorta of western diet-fed rabbit, atherosclerotic-prone mouse model as well as human patients with severe atherosclerotic lesions^14,15,28^. MIF itself can act as a ligand and binds to receptors, including CXCR2 and CXCR4, on the endothelial surface membrane to activate downstream inflammatory signaling pathways, leading to the recruitment of T cells and macrophages^29^. By blocking endothelial MIF with a monoclonal antibody, Schober *et al*. have reported a stabilized plaque phenotype with reduced foam cells and increased VSMC^30^. We show that endothelial cell-specific MIF can be up-regulated by pro-atherogenic OS, but down-regulated by LS which is intrinsically athero-protective (Fig. 2C,D), suggesting a potential early role in the process of atherosclerosis. Consistently, *in vivo*, in a rabbit model known to better recapitulate human atherosclerosis development^31,32^, and in the absence of additional risk factors beyond age, MIF expression is increased in regions of the aorta subjected to OS (pro-atherogenic) -lesser curvature and bifurcations, and virtually undetectable in areas subjected to LS (athero-protective), indicating that shear stress-dependent expression of MIF is an integral part of the initial stages of atherosclerosis and positively correlates with the known non-random development of atheroma in the aorta^10^.

Although it is known that MIF expression by inflammatory cells, such as macrophages, and by endothelial cells is increased in response to pro-inflammatory stimuli, the molecular components responsible of the transcriptional regulation of MIF are less understood^28,33^. Recent studies uncovered genetic polymorphisms in the promoter of MIF that are associated with the severity of carotid artery atherosclerosis^34^ and potentially cardiomyocyte response to ischemia^35^, and some polymorphisms show association to different aspects of cardiovascular disease, depending on the vascular bed affected^36–38^ or presence of additional risk factors^39^. Such findings highlight the need to understand the tissue- and context-specific mechanisms for transcriptional regulation of MIF. KLF2 is a key transcription factor involved in multiple biological processes, including inflammation and thrombosis. Most importantly, it is a master regulator induced by shear stress^40^. In addition to the regulation of eNOS, KLF2 is well-known anti-inflammatory factor in the endothelial cells that modulates thrombomodulin, plasminogen activator inhibitor, and cytokine-stimulated tissue factor^41^. In this study, KLF2, as expected, was found to have marked differential expression in ST vs LS, with significant higher expression in LS (Fig. 1A). We identified a KLF2 specific binding site in the MIF promoter (Fig. 3D) and shown that it is functional in inhibiting endothelial MIF mRNA expression by directly binding to the 5′-CACCC-3′ motif in the MIF promoter. Furthermore, knockdown of KLF2 potently overcame the LS-mediated inhibition of MIF, suggesting a natural athero-protective role of KLF2 in the endothelium. Interestingly, downregulation of KLF2 in ST conditions also lead to higher expression of the pro-inflammatory EC-specific MIF, consistent with KLF2 operating as a homeostatic modulator of MIF expression in endothelial cells, intrinsically protective against atherosclerosis. The actual extent of the effects of the KLF2/MIF axis remains to be addressed. One example could relate to the benefits of exercise^42^. Physical activity substantially reduces the risk of cardiovascular diseases. However, little is known about the underlying mechanisms. One intriguing hypothesis is that exercise increases the magnitude of shear stress to exert atheroprotective forces^43^, reasonably by up-regulating KLF2. On the other hand, reduction in heart rate is associated with increased switch to LS, thus potentially underlying the protective effects of sleep and beta-blockers through increase in KLF2 and reduced MIF^44^. The relative contribution of KLF2-dependent modulation of MIF to the overall KLF2 protective effects through eliciting a mild EC-inflammatory state in the OS regions of the aorta also remains to be determined^45^.

Overall, this work provides an easy access to the whole transcriptome pool and simplifies future research on novel targets for anti-atherosclerosis drug development. However, it is important to note that there are several limitations to this study. We used primary HCAECs at early passage for transcriptomic sequencing in response to different types of shear stress. Despite these HCAECs were appropriately cultivated and propagated, and were used for shear stress experiments at the same passage to minimize the artifacts from environment (e.g. culture condition) and preparation, ECs undergo significant culture-induced changes over propagation^46^, which could jeopardize their good approximation to HCAECs *in vivo*. Instead, a deep transcriptomic sequencing on both healthy HCAECs under LS and diseased HCAECs in the atherosclerotic lesions under OS would give us a more comprehensive understanding of the biomechanical impact on endothelial biology during atherogenesis. Temporal factor is another concern. Exposure time to shear stress is essential in signaling pathway activation and subsequent gene expression. We exposed HCAECs to different types of shear stress for 24 hours, however, others have reported that transient or short-term exposure to shear stress induces a different set of gene expression^22,47^. It would be of great interest to profile the endothelial transcriptome at different time points to yield a comprehensive understanding of the dynamic change in ECs in response to shear stress. Another limitation of this study is that the library preparation and RNA-seq kit used generates a 50-nucleotide (nt) paired end and assemble a >200 nt read, which doesn’t include the shear stress-induced changes of microRNAs, the size of which is approximately 23 nt^48^. The function and regulation of known mechanosensitive microRNAs have been reviewed^49,50^. However, it will be very useful to obtain the microRNA profile in ECs under different shear stress. On the other hand, the unexpected result that static cultures have an overall inflammatory profile intrinsically closer to that of EC subjected to oscillatory stress was somehow surprising^21^. This finding bears consideration for *in vitro* studies involving static cultures and relating to the EC inflammatory response while creating some questions regarding their translatability and correlation to the EC biology in areas of the aorta that are under LS as well as in the *in vitro* studies of therapeutic potential.

In conclusion, endothelial MIF and KLF2 play a critical role in the initiation and progression of atherosclerosis. The newly uncovered shear stress-sensitive characteristic of EC-specific MIF together with the novel finding that KLF2 directly inhibits MIF expression at the transcriptional level contributes to deepening our understanding of atherogenesis from a biomechanical perspective.

## Methods

Primary antibodies against β-actin (sc-1616), KLF2 (sc-28675), MIF (sc-20121), VE-cadherin (sc-6458), were purchased from Santa Cruz Biotechnology (Santa Cruz, CA, USA). Primary antibodies against KLF11 (M01, clone 8F4) and KLF4 (12173, D1F2) were obtained from Abnova (Walnut, CA, USA) and Cell Signaling Technology (Danvers, MA, USA), respectively. Primary antibodies against MIF (MAB289-100) for immunocytochemistry were purchased from R & D System (Minneapolis, MN, USA). Secondary Antibodies donkey anti-mouse IgG and donkey anti-goat IgG were purchased from Jackson ImmunoResearch (West Grove, PA, USA). Lipopolysaccharide (LPS) of *Escherichia coli* was purchased from Sigma-Aldrich (St. Louis, MO, USA). Adenovirus Ad-GFP-hKLF2, Ad-GFP-U6-hKLF2-shRNA, Ad-GFP-U6-scrambled were purchased from Vector Biolabs (Malvern, PA, USA).

### Cell Culture

Human coronary artery endothelial cells (HCAECs) (Cell Applications, San Diego, CA, USA) were cultured in MesoEndo Cell Growth Medium with 5% CO_2_ at 37 °C in a cell culture incubator. Human umbilical vein endothelial cells (HUVECs) (Lonza, Alledale, NJ, USA) were cultured with M199 medium (Invitrogen, Carlsbad, CA, USA) containing 16% fetal bovine serum (FBS), 1 ng/ml recombinant human fibroblast growth factor (Sigma-Aldrich, St. Louis, MO, USA), 90 μg/ml heparin and 20 mM HEPES. HCAECs for RNA-sequencing purposes were obtained at passage 2 and used for shear stress experiments at passage 6 and HUVECs within 4 passages were used in the follow up experiments. AD-293 (ATCC, VA, USA) cells were cultured in DMEM with 10% FBS.

### Shear Stress Studies and RNA Sequencing Experiment

A detailed protocol of shear stress experiments has been described previously by our lab^21^. Briefly, ECs at 90% confluence in 100-mm tissue culture dishes were exposed to 15 dyn/cm^2^ unidirectional LS, bidirectional OS at 1 Hz cycle (±5 dyn/cm^2^) by rotating a Teflon cone (0.5° cone angle) with a programmed stepping motor (Servo Motor), or static cultured condition (ST) for 24 hours (n = 4, respectively). RNA-seq fastq files were aligned to the current version of the human genome primary assembly using the Subread program and the GenCode v24 annotation was used for summarizing read counts on each gene. The limma package along with the voom transformation was used for differential expression analysis between different types of shear stresses. P values were adjusted by default Benjamini-Hochberg procedure. The cutoff requires at least 1 in 4 samples of one shear stress condition with expression level ≥1 rpm (one count per aligned million reads) for a gene to be included in the analysis to increase the sensitivity of detecting differentially expressed genes.

### Plasmid Construction, Transformation and Viral Infection

A human MIF gene promoter fragment (−1053 ~ +24 bps) containing the predicted KLF2 core binding motif (CACCC) was cloned from genomic DNA and inserted into the pGL4.20 luciferase reporter vector (Promega, Madison, WI, USA). Mutation of the putative KLF2 binding motif (CATTC) was generated using the Site-Directed Mutagenesis kit (New England Biolabs, MA, USA). AD293 cells were co-transfected with these plasmids plus pRL-TK *Renilla* luciferase control reporter vector at 80% confluence with Lipofectamine 2000 (Invitrogen, CA, USA) in Opti-MEM (Life Technologies, CA, USA) for 4 hours followed by 48-hour incubation in DMEM with 10% FBS. Dual-luciferase activities were detected with a dual-luciferase reporter assay in accordance with the recommended protocol and all data were normalized to *Renilla* luciferase activity (Promega, Madison, WI, USA). For adenovirus infection, confluent ECs were infected with targeted adenovirus for 2 hours followed by fresh medium incubation. Infected ECs were then used for experiments after 48 hours.

### En Face Staining

All animal experiments were conducted according to protocols approved by Institutional Animal Care and Use Committee at the University of Michigan. New Zealand white rabbits (15~20 months old, fed a normal chow diet, n = 4) were euthanized and immediately perfused with cold phosphate buffered saline (PBS) containing 5% heparin followed by cold fresh 4% paraformaldehyde (PFA) solution. Aortas were carefully dissected and fixed in 4% PFA for another 20 minutes. After wash with cold PBS, they were neutralized with 100 mM glycine, aortas were permeabilized with 0.2% Triton X-100 and then incubated in blocking solution (75 mM NaCl, 18 mM Trisodium citrate, 4% FBS, 1% bovine serum albumin (BSA), 0.05% Triton X-100) for 2 hours followed by incubation with primary antibodies diluted in serum-reduced blocking solution (2% FBS) at 4 °C for 48 hours. Aortas were then washed in washing solution (75 mM NaCl, 18 mM Trisodium citrate, and 0.05% Triton X-100) for 1 hour and subsequently incubated with secondary antibodies for 1 hour at room temperature in the dark. Aortas were washed, mounted with Prolong Gold with DAPI (Molecular Probes, OR, USA) and imaged using a Nikon A1 confocal microscope.

### Chromatin Immunoprecipitation Assays (ChIP)

ChIP assays were performed using the EZ-ChIP Kit (EMD Millipore, MA, USA) according to the manufacturer’s instruction. Briefly, HUVECs were first cross-linked with 1% formaldehyde for 10 minutes followed by glycine solution addition. Chromatin extracts were harvested and sonicated into 200–1000 bp DNA fragments on ice. DNA fragments were then preabsorbed with protein G-agarose, centrifuged and incubated at 4 °C with anti-KLF2 antibody (Santa Cruz Biotechnology, CA, USA) or normal anti-rabbit IgG overnight. After incubation with protein G-agarose at 4 °C, the immunoprecipitated complexes were washed in low-salt buffer, high-salt buffer, LiCl buffer, and Tris-EDTA buffer in sequence. The DNA-protein crosslinks were reversed by overnight incubation at 65 °C with subsequent proteinase K digestion for 1 hour at 45 °C. Purified DNA was later collected and used for real-time quantitative PCR. The primers used for the analysis of MIF promoter are in Supplemental Table 1.

### RNA preparation and qPCR analysis

Total RNA was extracted from endothelial cells using RNeasy Kit (QIAGEN, Hilden, Germany) and reverse-transcribed into cDNA with SuperScript III kit (Thermo Fisher Scientific). RNA quantification was determined by qPCR (BioRad, Hercules, CA), using iQ SYBR Green Supermix (BioRad, Hercules, CA). The mRNA abundance was normalized to internal control 18 S or HPRT1 (hypoxanthine phosphoribosyltransferase 1), unless otherwise indicated. The primers used are shown in Supplemental Table 1.

### Western Blotting

Confluent ECs were washed with cold PBS and lysed with Pierce RIPA buffer (Thermo Scientific, Rockford, lL, USA) containing protease inhibitors (Roche Applied Science, Indianapolis, IN, USA). Cell extracts were centrifuged and the supernatant was mixed with loading buffer. In general, 25 μg of protein samples were loaded per lane on a 15% SDS-PAGE gel and underwent gel electrophoresis followed by transfer to 0.2 μm nitrocellulose membrane (Bio-Rad, Hercules, CA, USA) at 4 °C. Membranes were blocked for 1 hour at room temperature and incubated with primary antibodies at 4 °C overnight. After washing, membranes were incubated with a diluted (1: 10,000) IRDye-conjugated secondary antibody (LI-COR Biotechnology, Lincoln, NE, USA) for 1 hour at room temperature. Membranes were scanned and the intensity of the protein bands was quantified using Image Studio software (LI-COR).

### Statistical Analysis

Statistical significance was assessed by unpaired Student *t* test between 2 groups and one-way ANOVA with Bonferroni post hoc test among 3 groups or more. A value of *p < *0.05 was considered statistically significant. All results are representative from at least 3 independent experiments. Data are presented as mean ± SEM.

## Electronic supplementary material

Supplementary information
