# Supplementary material for: Laminar Flow Attenuates Macrophage Migration Inhibitory Factor Expression in Endothelial Cells
Source: Sci Rep. 2018 Feb 5;8:2360. doi: 10.1038/s41598-018-20885-1 (PMC5799263; doi:10.1038/s41598-018-20885-1)

Laminar Flow Attenuates Macrophage Migration Inhibitory Factor Expression in Endothelial Cells

Congzhen Qiao[1, 2], Shengdi Li[3, 4], Haocheng Lu[5], Fan Meng[6], Yanbo Fan[5], Yanhong Guo[5], Y. Eugene Chen[1, 5, *], Jifeng Zhang[5, *]

Affiliations:

1. Department of Pharmacology, University of Michigan Medical Center, Ann Arbor, Michigan, 48109, USA
2. Shanghai Jiao Tong University School of Medicine, Shanghai, 200025, China
3. Key Lab of Computational Biology, CAS-MPG Partner Institute for Computational Biology, Shanghai Institutes for Biological Sciences, Chinese Academy of Sciences, Shanghai, 200031, China
4. University of Chinese Academy of Sciences, Beijing, 100049, China
5. Frankel Cardiovascular Center, Department of Internal Medicine, University of Michigan Medical Center, Ann Arbor, Michigan, 48109, USA
6. Department of Psychiatry and Molecular and Behavioral Neuroscience Institute University of Michigan Medical Center, Ann Arbor, Michigan, 48109, USA

* Correspondence and requests for materials should be addressed to JZ (email: jifengz@umich.edu) or YEC (email: echenum@umich.edu)

Supplementary table 1. Primers used for PCR

Gene Name	Species	Forward	Reverse
**KLF2**	human	5'-CTACACCAAGAGTTCGCATCTG-3'	5'-GTGCCGTTTCATGTGCAG-3'
**ACTB**	human	5'-TGACATTAAGGAGAAGCTGTGC-3'	5'-TGGAGTTGAAGGTAGTTTCGTG-3'
**MIF**	human	5'-ATGTTCATCGTAAACACCAACG-3'	5'-CTTGCTGTAGGAGCGGTTCTG-3'
**HPRT1**	human	5'-TCGAGATGTGATGAAGGAGATG-3'	5'-TTTATGTCCCCTGTTGACTGGT-3'
**18S**	human	5'-GGAAGGGCACCACCAGGAGT-3'	5'-TGCAGCCCCGGACATCTAAG-3'
**ChIP-MIF-Promoter**	human	5’-TTGTCCTCTTCCTGCTATGTCA-3’	5’-GGTAAACTCGGGGACCATCTA-3’
**ChIP-MIF-Neg-ctrl**	human	5'-TCATCTTCAAAGCCGGTAATG-3'	5'-AGGGAGATTAGCATGGAGCAT-3'

Supplemental figure 1. Whole blot for Figure 2B

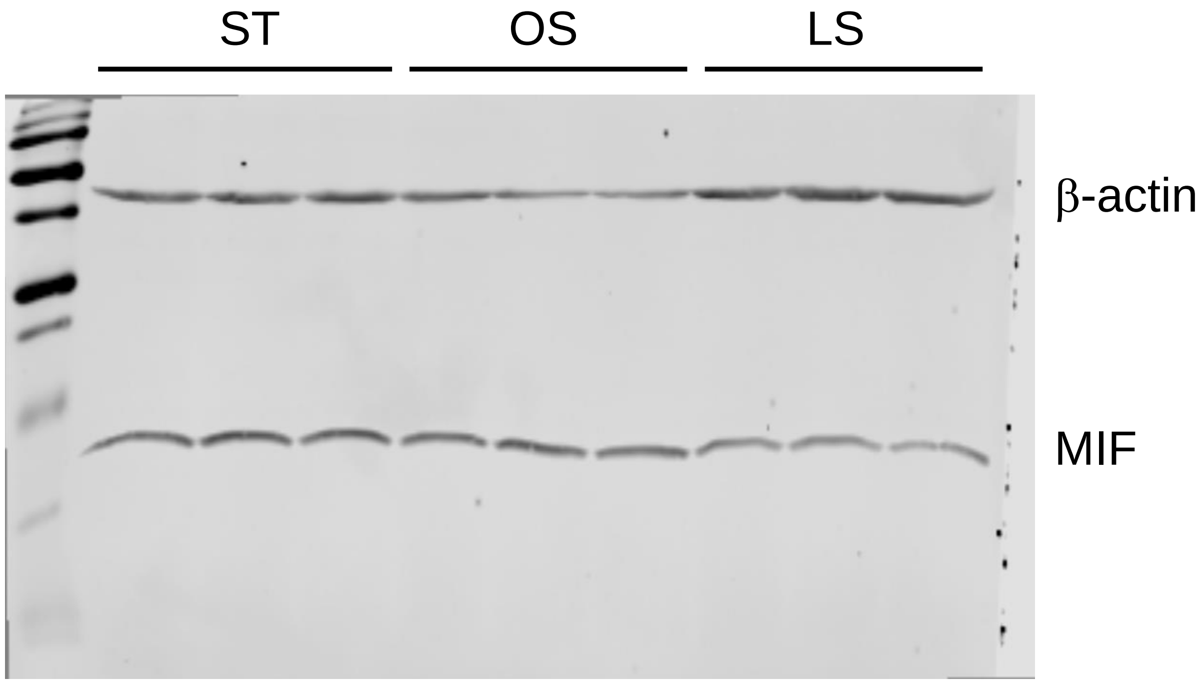

Supplemental figure 2. Whole blot for Figure 3C

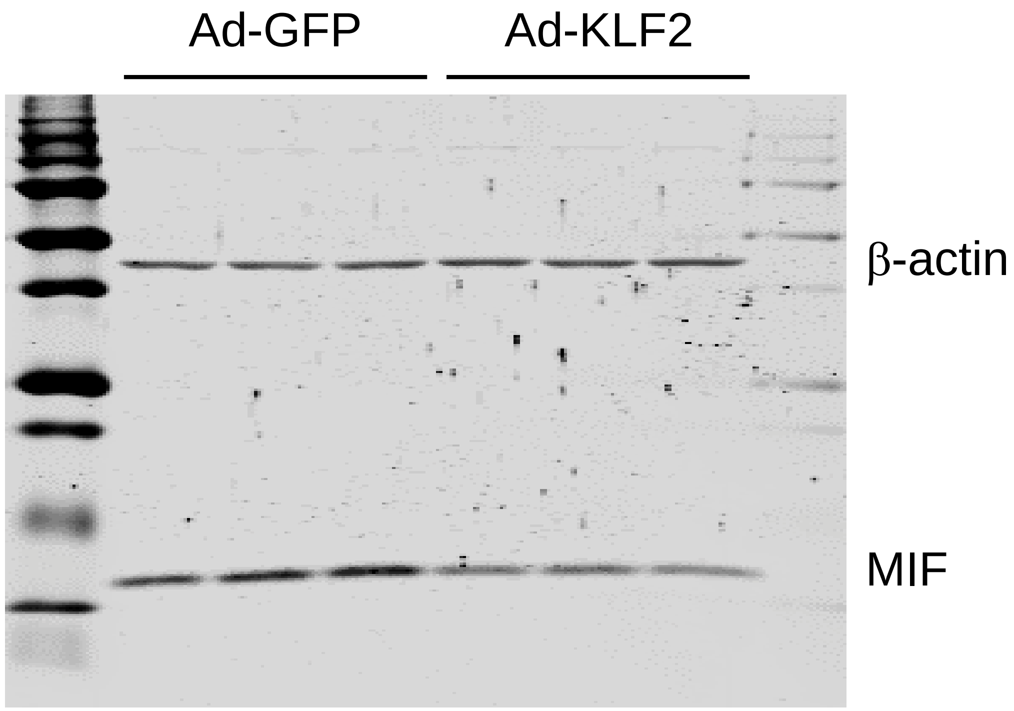

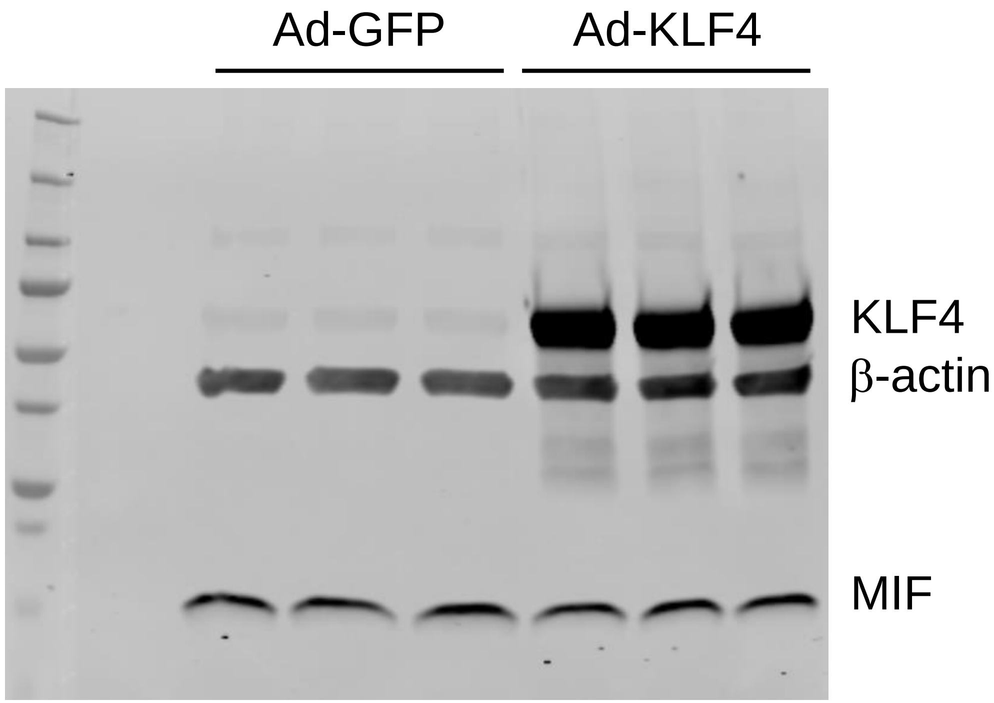

Supplemental figure 3. Whole blot for Figure 3C

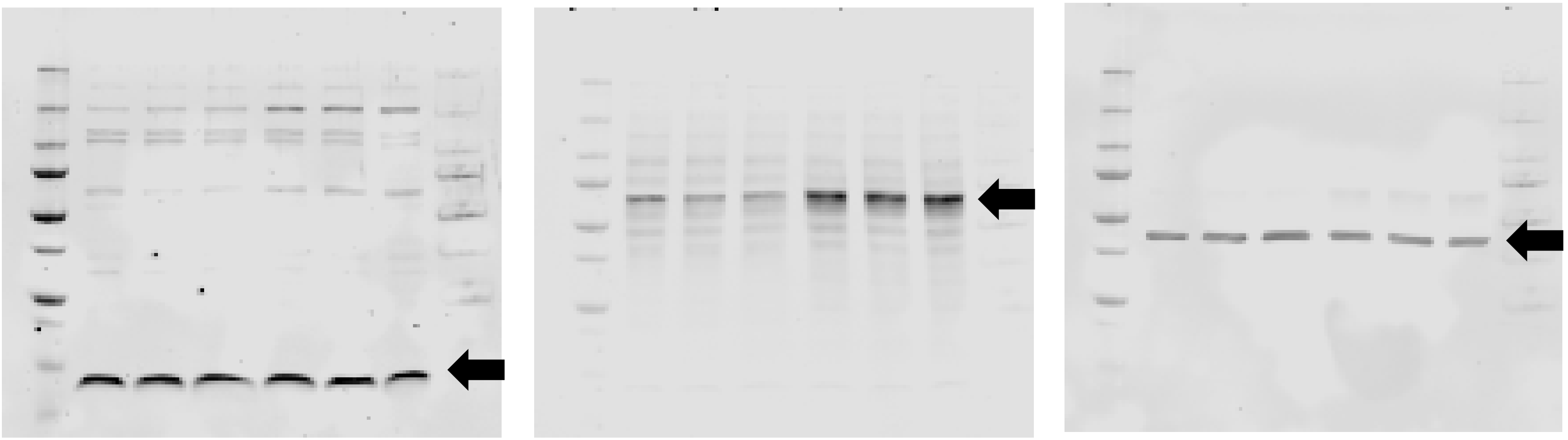

MIF KLF11 β-actin

Supplemental figure 4. Whole blot for Figure 4C

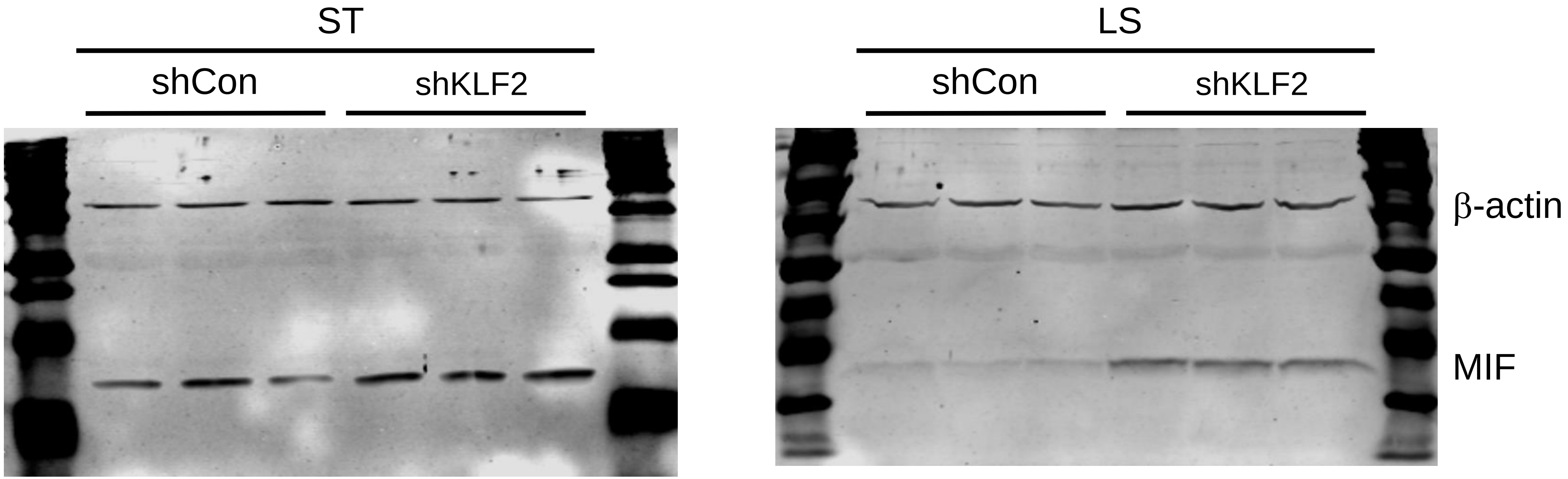

Supplement: Supplementary file 1 — Supplementary information [file 41598_2018_20885_MOESM1_ESM.pdf]
